# Informative Wavelength Selection for Evaluation of Bacterial Spoilage in Raw Salmon (*Salmo salar*) Fillet Using FT-NIR Spectroscopy

**DOI:** 10.3390/foods14122074

**Published:** 2025-06-12

**Authors:** Roma Panwar, Shin-Ping Lin, Shyh-Hsiang Lin, Jer-An Lin, Yu-Jen Wang, Yung-Kun Chuang

**Affiliations:** 1 Ph.D. Program in School of Nutrition and Health Sciences, College of Nutrition, Taipei Medical University, Taipei 11031, Taiwan; da07112005@tmu.edu.tw; 2 School of Food Safety, College of Nutrition, Taipei Medical University, Taipei 11031, Taiwan; splin0330@tmu.edu.tw (S.-P.L.); lin5611@tmu.edu.tw (S.-H.L.); 3 Master Program in Food Safety, College of Nutrition, Taipei Medical University, Taipei 11031, Taiwan; 4 Graduate Institute of Food Safety, National Chung Hsing University, Taichung 40227, Taiwan; lja@nchu.edu.tw; 5 Department of Food Science and Biotechnology, National Chung Hsing University, Taichung 40227, Taiwan; 6 Department of Radiation Oncology, Fu Jen Catholic University Hospital, New Taipei City 24352, Taiwan; 138697@mail.fju.edu.tw; 7 School of Medicine, College of Medicine, Fu Jen Catholic University, New Taipei City 242062, Taiwan; 8 Department of Radiation Oncology, Shuang Ho Hospital, Taipei Medical University, New Taipei City 23561, Taiwan; 9 Nutrition Research Center, Taipei Medical University Hospital, Taipei 11031, Taiwan

**Keywords:** salmon, Fourier-transform near-infrared spectroscopy, total bacterial count, spoilage, informative wavelengths

## Abstract

This study highlights the potential of Fourier-transform near-infrared (FT-NIR) spectroscopy for the on-site, nondestructive detection of spoilage caused by bacterial action in raw salmon (*Salmo salar*) fillets. A stepwise multiple linear regression model with first-derivative spectrum transformation was combined with the standard normal variate and detrend preprocessing techniques. The model achieved correlation values of 0.97 in both the calibration and validation sample sets, with root mean square error values of 0.18 and 0.20 log CFU/mL, respectively. These accurate results reveal the precision of FT-NIR spectroscopy for assessing the spoilage caused by bacteria. The most informative wavelengths (885.27 nm, 1026.27 nm, 1039.93 nm, 1068.38 nm, 1257.55 nm, 1267.75 nm, and 1453.49 nm) related to the total bacterial count’s identification were obtained. The innovative, cost-effective, and feasible approach outlined in this article is a promising methodology for enhancing the safety and quality standards of various fishery products.

## 1. Introduction

Food safety and quality are imperative for human safety, health, and social progress [1]. Annually, millions of people become sick due to unsafe food, and hundreds of thousands die as a result [2]. One-quarter of the global food supply is wasted solely because of spoilage due to microbial action [3]. This is a serious threat to food security and a major obstacle to achieving sustainable development goals. Food safety can be improved through two major factors: (1) food safety regulations and (2) improved technological means of rapidly detecting food-borne pathogens.

Aquaculture is a method to feed growing populations and prevent malnutrition. It produces highly nutritious fish and other seafood products, such as salmon, which are rich in the omega-3 fatty acids, vitamins, proteins, and minerals considered a crucial part of a healthy diet. The salmon supply chain is the most efficient in the world [4], and salmon farming is growing far more quickly than is aggregate aquaculture production [5]. However, salmon is highly perishable [6]; and with the increased demand for seafood products, the safety of this nutritious food has become a matter of concern. Endogenous enzymes and psychrophilic bacteria are abundant in such products because of their fragile structure and highly nutritious composition; both preharvest and post mortem variables can affect the value and safety of fish products [7]. Spoilage is attributable to bacterial action for most seafood products, and although factors such as autolysis and chemical oxidation can also contribute to spoilage [6], bacteria always act as a catalyst that accelerates on-going spoilage. Hence, evaluating fish for their quality or bacterial growth is critical to the growth of the aquaculture industry.

Salmon is consumed in various forms, including raw as sushi or in other semi-processed forms; these products can all be easily contaminated by bacteria from both environmental and human reservoirs. Even cold-smoked salmon, which is a ready-to-eat food with a long shelf life that is widely eaten cold, may be contaminated with bacteria during processing, distribution, and storage [8]. Earlier research showed that processing steps can result in the contamination of salmon fillets with pathogens like *Pseudomonas* and *Shewanella* [9]. In seafood products, spoilage progresses fast and is caused by microbial and enzymatic activity that changes the chemical composition in products. FT-NIR enables the nondestructive and rapid detection of such changes via spectral changes associated with lipid breakdown, proteolysis, and water content variations. Conventional methods for detecting spoilage and bacteria in salmon fish, such as culturing, pour-plating or spread-plating, and sensory evaluation, are generally time-consuming, destructive, and labor-intensive. Although culture-based methods (i.e., plating and enumeration) are widely used to quantify bacterial populations, these methods require lengthy incubation periods; it is often several days before results can be obtained. The reliance on human perception in sensory evaluations limits its sensitivity and reproducibility; it is poorly suited for detecting spoilage at an early stage. Sensory methods are expensive, lengthy, and difficult to standardize [10]. Moreover, both culture-based methods and sensory evaluation are destructive sampling approaches and are thereby inapplicable for real-time monitoring. Sensory evaluation involves the tasting, smelling, and handling of products. These actions alter the samples, which means it cannot be reused for any purpose. Additionally, advanced techniques like high-performance liquid chromatography (HPLC) and gas chromatography (GC) require gases, chemicals, and solvents in order to operate and are not very environmentally friendly. All of these drawbacks highlight the need for alternative, nondestructive approaches like FT-NIR for detecting salmon spoilage that are rapid, accurate, and objective. Previous studies indicated that FT-NIR can detect spoilage-related changes in complex food matrices, including fish and shellfish, through spectral variations utilizing a chemometric approach [11]. FTNIR possesses the ability to penetrate deeper into food samples, which offers advantages over surface-restricted techniques, allowing a more accurate spoilage assessment in food products with complex food matrices.

Near-infrared (NIR) indicates a range of wavelengths between the mid-infrared and visible regions (4000–12500 cm^−1^). NIR spectroscopy can be combined with the Fourier transform (FT) in the form of FT-NIR spectroscopy; this enhances the spectral resolution and signal-to-noise ratio of NIR spectroscopy, improving its analytical precision [12]. NIR spectra can be interpreted as the absorbance and wavenumbers or wavelengths of a material. These spectra contain peaks that represent the structural and molecular characteristics of the sample. Specifically, these peaks represent the stretching and vibrations of various functional groups, such as those of O–H (which are byproducts of alcohol groups and can indicate the presence of water), S–H (sulfides), N–H (amines and proteins), C–H (carbohydrates), and R–C–R′ (ketones). Observing these functional groups through the NIR spectroscopy of fish can reveal the onset and progression of spoilage by identifying biochemical changes [7]. Therefore, chemometric methods can be used to validate the use of NIR to nondestructively identify fish spoilage. Alexandrakis et al. (2012) reported the successful use of NIR and mid-infrared spectroscopy to determine the microbial spoilage of chicken, defined as a total viable count (TVC) on chicken breast fillets, whilst classifying day 8 and day 14 samples (9.61 to 10.37 log_10_ CFU/g) [13]. Moscetti et al. (2014) [14] identified moldy chestnuts with NIR in their research. They observed a good recognition with a 97% classification accuracy between medium and heavily fungus-infected and control chestnuts. Moreover, the application of NIR with hyperspectral imaging (HSI) gained wide attention due to their combined ability to provide spectral and spatial information by integrating conventional spectroscopy and digital imaging. It helps in the quantification and visualization of bacteria in different food products [11]. One of these articles focused on NIR spectral regions to identify the TVC determination from salmon flesh [15]. NIR hyperspectral imaging (NIR-HSI) in the range of 900–1700 nm has shown promising results for the determination of *Enterobacteriaceae* in salmon flesh during cold storage [16]. NIR-HSI has been also used to evaluate the total counts of *Enterobacteriaceae* and *Pseudomonas* spp. on edible salmon flesh [17]. However, HSI requires high-resolution detectors and complex optical components, which significantly increase its overall cost as compared to FT-NIR spectroscopy. Furthermore, Vis/NIR (visible and near-infrared) spectroscopy in the 400 to 1000 nm range with time series hyperspectral imaging method (TS-HSI), combined with partial least square regression (PLSR), was successful in obtaining informative wavelengths for the TVC distribution in salmon chops [15]. However, TS-HSI produces high-dimensional spectral data compared to FT-NIR, which is challenging to interpret without advanced machine learning methods [15]. On the other hand, visible spectroscopy is highly influenced by external factors and only interacts with the surface of samples, unlike FT-NIR, which possesses the ability to penetrate deep into the sample and provide a bulk analysis of products. In a short communication, FT-NIR spectroscopy was also reported to predict bacteria content in salmon [18]. Although that study obtained a calibration equation with an R^2^ = 0.95 and root mean square error (RMSE) of 0.18 log CFU/g, the error of the validation curve was large (R^2^ = 0.64 and RMSE = 0.32 log CFU/g). Thus, researchers must refine this methodology before it can be considered reliable for practical applications. Overall, FT-NIR spectroscopy possesses potential for the monitoring of microbial growth and the progression of spoilage in fish. Some recent studies published between 2020 and 2024 demonstrate the expanded application of NIRS and NIR-HIS combined with various chemometric and machine learning methods for the quality analysis of different products [11]. In the field of qualitative analysis, NIRS was used to identify origin-related fraud using its spectral fingerprinting feature in salted ripened anchovies with a good predictive accuracy [19]. Additionally, with regard to quantitative analysis, NIRS combined with PLSR was used to predict the textural properties of silver carp with improved performance (Rp^2^ values between 0.83 and 0.95) [20]. Through an analysis of the spectral fingerprints associated with microbial metabolites and biochemical changes, FT-NIR spectroscopy can provide real-time insights into the spoilage and total bacterial count (TBC) of fish samples. Moreover, a few studies also demonstrated the application of NIR with an optimized wavelet neural network model for assessing histamine levels in mackerel, with acceptable prediction results (R^2^p of 0.79) [21], and the feasibility of NIRS along with PLSR for determining multiple quality attributes simultaneously, such as total volatile basic nitrogen (TVB-N), thiobarbituric acid (TBA), and whiteness in vacuum-packaged catfish fillets, with accuracies (R^2^) ranging from 0.86 and 0.90 [22]. More recent research between 2021 and 2023 further expanded NIR-HSI applications, including the successful prediction of TVB-N content in tilapia fillets (R^2^p = 0.8524) [23] and rainbow trout fillets (R^2^p = 0.853) [24], often incorporating the deep learning method. Whilst there have been advancement in NIRS and HIS-NRI, these are, however, primarily applied to other purposes with different methods. The objective of the present research was to investigate the efficacy of combining FT-NIR spectroscopy with chemometric approaches (SMLR and PLSR) for the nondestructive determination of the TBC in salmon and to identify suitable wavelengths that correspond to the presence of bacteria.

## 2. Materials and Methods

### 2.1. Sample Procurement and Preparation

Raw Norwegian salmon fillets were purchased from a supermarket in Taipei, Taiwan. The fillets were then directly taken to a laboratory of Taipei Medical University and divided into 10 g, 3 cm cubes in sterile conditions. These samples were divided into two groups: fresh and spoiled (100 samples in each group). On the day of purchase (we indicated it as day 0), the fresh samples were packed separately in sterilized bags and underwent FT-NIR spectroscopy measurement followed by microbiological analysis; however, to facilitate the natural process of spoilage, we stored another group of fresh samples at room temperature (28 °C for 4 days; 0, 1, 2, 3 days) on a laboratory desk. A storage temperature of 28 °C was chosen on purpose to speed up the spoilage process, as it is commonly applied in spoilage studies to facilitate microbial and enzymatic activity under controlled conditions. We categorized this group as “spoiled sample groups”. Spoiled salmon samples were taken for FT-NIR spectroscopy measurements on day 4, followed by microbiological analysis.

### 2.2. NIR Measurements

Spectroscopy measurements were performed using an MPA 11 analyzer FT-NIR spectrometer (Bruker Corporation, Billerica, MA, USA). First, a background scan was performed. A 10 g sample was then placed in a sterile glass sample holder. Moisture from the sample was absorbed with sterile tissue paper, and the sample was covered with a golden reflector for spectral analysis. The signal values were validated after every sample scan. This process was performed for all the samples, with a setting of 32 sample scans and 64 background scans to enhance the signal-to-noise ratio in order to obtain reliable spectral data. All scans had a resolution of 8 cm^−1^, as it provides a good balance between minimizing noise and obtaining sufficient spectral detail. After each spectral analysis, the glass holder was cleaned and sanitized. The results were obtained in the form of absorbance [log(1/*R*)] and wavenumber (cm^−1^), where *R* is the reflectance. Values were exported to an Excel file for analysis.

### 2.3. Microbiological Analysis

After the FT-NIR spectroscopy experiment, microbiological testing was performed through conventional plating and colony counting. Nutrient agar (NCM0269, Neogen Culture Media, Heywood, Lancashire, UK) was used to prepare a medium for bacterial growth. The medium was autoclaved using a high-pressure steam sterilizer autoclave (Tuttnauer, Breda, Noord-Brabant, The Netherlands), and the melted medium was poured onto sterile polystyrene Petri plates (Biologix Group Limited, Jinan, China). Plates with solidified medium were used for the inoculation of cultures from the fresh and spoiled salmon samples, and the TBC was quantified for each. After each culture was inoculated, the inoculum was uniformly spread over a solidified Petri plate. The plates were incubated at 37 °C for 48 h in an LM-420D shaking incubator (Yihder Technology Co., Ltd., New Taipei City, Taiwan). Incubation at 37 °C facilitates the optimal growth of mesophilic bacteria, including potential human pathogens and common hygiene or spoilage indicators, as it reflects conditions suitable for bacterial growth in food products. After incubation, the colonies were counted, and the results were converted into the logarithm of colony-forming units per milliliter (CFU/mL) as the number of colonies multiplied by the dilution factor and divided by the volume of the culture plate.

### 2.4. Data Analysis

Statistical analysis was conducted using MATLAB 2013b (The MathWorks, Inc., Natick, MA, USA) and WinISI II v1.02a project managing software (Infrasoft International, State College, PA, USA). The NIR wavelength range for the analysis was 11,536 to 3952 cm^−1^. PLSR and stepwise multiple linear regression (SMLR) was applied to explore the relationships between the reflectance spectra and the concentrations of the target constituent (i.e., TBC). Three widely used preprocessing approaches, including standard normal variate (SNV) transformation, detrend, and multiplicative scatter correction, were applied to select the most relevant method to correct light scattering prior to using the spectra in the calibration model. Among these approaches, SNV is mainly used to address the multiplicative and additive effects caused by scattering and particle size variability in the raw spectra [25]. It normalizes the spectrum data whilst removing overall scaling or gain effects. Detrend combined with the SNV facilitates wavelength-dependent scattering effects via fitting a second-order polynomial to each spectrum. It improves the wavelength dependency by removing the curvilinearity and baseline shift of the spectra [25]. Additionally, multiplicative and additive spectral corrections are applied to perform corrections in the original spectra that correspond to the mean spectrum [26]. After performing a light-scattering correction, the spectra of the salmon samples were subjected to three independent treatments: (1) smoothing; (2) smoothing with the first derivative; and (3) smoothing with the second derivative. These treatments were applied to choose the best pre-treatment parameters, including the segments and derivative gap (range, 2–20), with the gap being equal to the segments. Derivative transformation was applied to enhance the resolution of overlapping peaks, which is not possible via raw data. First-derivative transformation improved the quantification and enhanced the resolution of the obtained spectra by resolving overlapping peaks and removing baseline shifts. It is also crucial to increase accuracy for quantitative measurements. Second-derivative transformation provides similar advantages over the raw spectrum but sometimes lead to the distortion of data. In our study, the error rate was quite high after applying second-derivative transformation; hence, we performed the further analysis with first-derivative transformation. Smoothing reduces noise in the spectral data whilst maintaining the overall shape of the spectrum. For comparative analysis, it is necessary to keep the smoothing equal to the derivative gap as a consistent approach for data processing. Furthermore, our analysis included the following main steps: (1) spectral pre-treatments; (2) selection of specific wavelength regions; (3) generation of equation files; (4) elimination of outliers; and (5) determination of the best calibration and validation model. Three-fold cross-validation was employed in order to facilitate the objective selection of the parameters (steps 1 and 2). Grouping (step 3) was performed to assess the predictability of the calibration models built by the PLSR and SMLR model in terms of quantitative approach for prediction [27]. All samples were examined for outliers. There were 32 outliers, which were eliminated before generating the equation files. The remaining data were divided into validation and calibration sample sets in a ratio of 1:2. Of the 100 samples used for TBC analyses, the results for 68 samples were valid for further analyses. Validation is crucial to ensure the prediction capability of any model, and it provides confidence about the model [28]. The obtained laboratory values (log CFU/mL) were divided such that examples with high and those with low TBC values were present in both the calibration and validation sample sets. Spectral calibration equations of SMLR and PLSR were constructed using WinISI software to predict the TBCs, and the best-performing equation (highest R^2^ and lowest RMSE) was applied to the calibration and validation datasets to predict the TBCs.

The model with the best results was SMLR combined with SNV and detrend (SNV followed by detrending). SMLR is a multiple linear regression combined with variable selection scheme, which facilitates the quantification of association between dependent and one or more independent variables [29]. It is an effective method for obtaining results from relevant variables, such as measurements of specific wavelengths instead of an entire spectrum. It follows selection and elimination steps until the addition of new variables results into no further improvement in the model [30]. Furthermore, comparative analysis was performed using PLSR. As compared to SMLR, PLSR is ideally used for multicollinearity issues and has been widely used for quantitative analysis in food sectors. PLSR possess the ability to combine the advantages of linear regression analysis and PCA [31]. It is more suitable when the number of samples is smaller than the number of variables [32]. Additionally, PCA was performed with MATLAB software to identify differences between both the spoiled and fresh sample groups. PCA is a method of identifying primary components (PCs) that explain the greatest variation in the data. PCA is a widely used method for latent variable projection [33] and can enable various data visualizations, such as score plots and graphs. Score plots can show clusters, patterns, and outliers in data [34]. Similarly, variables can be represented as loading plots that further help in understanding covariances and differences between groups. Each model was evaluated using the following metrics: the correlation coefficient for the calibration set (RC), root mean square error of calibration set (RMSEC), correlation coefficient for the validation set (RV), and root mean square error of validation set (RMSEV). Models with larger correlation coefficients R and a lower RMSE were considered to have a better performance. RMSE was calculated using the equation below:

(1) $$RMSE=\frac{\sqrt{\sum \left(P_{i}-O_{i}\right)2}}{n}$$

where *P_i_* represents the predicted values, *O_i_* denotes the observed values, and *n* is the total number of observations.

## 3. Results and Discussion

### 3.1. NIR Spectroscopy and Bacterial Characteristics of Raw Salmon Fillets

**Figure 1:**
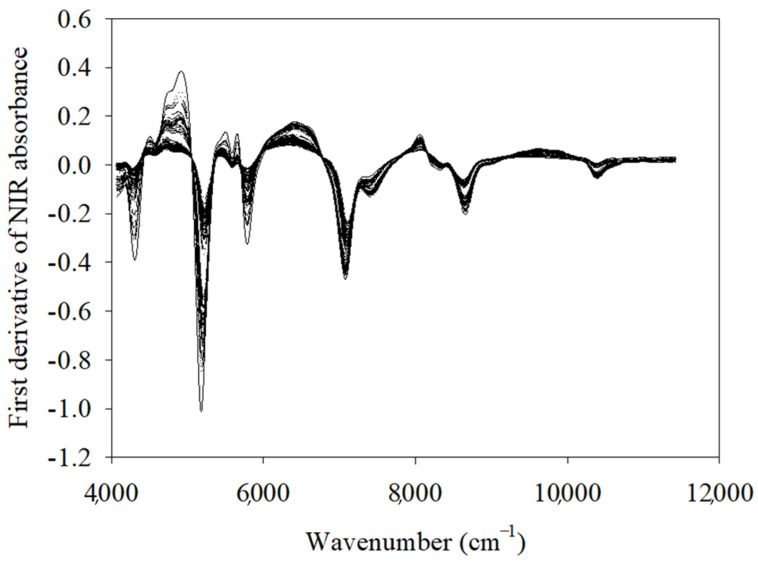
NIR spectra of salmon fillets—the first-derivative spectra.

The TBC analysis (measured TBC values) revealed a wide range of degrees of bacterial growth among the spoiled samples; the counts ranged from 7.46 to 9.79 log CFU/mL. Bacterial counts more than 9 log CFU/mL are above typical spoilage thresholds, but they were included to monitor the full progression of spoilage under accelerated conditions. Focusing on these advanced stages helps in understanding the microbial activity and quality deterioration at later stages and to ensure that detection methods remain reliable across the entire spoilage process. It helps in strengthening the validity of detection methods. By contrast, the fresh samples had TBC levels consistently below 5.7 log CFU/mL; this aligns with previous findings [18]. Figure 1 presents the first-derivative transformation of the NIR spectra; this transformation was useful for identifying spoilage-related changes in the salmon samples. Its spectral peaks revealed alterations in molecular vibrations within functional groups, highlighting the effects of spoilage processes on the chemical composition of the fillets. Peaks corresponding to protein, amide, and carbohydrate functional groups were observed in different overtone regions, emphasizing that bacterial metabolism produces spectral features due to the lipid degradation, proteolysis, and production of exopolysaccharides. Hence findings in our study indicate that FT-NIR spectroscopy can detect and monitor spoilage in fish products through bacterial metabolism.

### 3.2. Optimization of Spoilage Identification Method

Although Table 1 reveals that suitable results were obtained with the first-derivative transformation in the wavelength range 867–2505 nm via SMLR, the method was optimized for spoilage identification through parameter selection. The PLSR method was used for a comparative analysis, and the results are shown in tabular form for both the models (Table 1 and Table 2). The selected parameters that yielded optimal results for the detection of bacterial spoilage were the first-derivative transformation with smoothing values and derivative gaps as 2, 2, respectively. Second-derivative transformation with other combinations of smoothing values and derivative gaps did not result in equation files with a high R^2^ and lowest RMSE values. Additionally, the PLSR model with similar parameters resulted in a wide range of wavelengths, and it was difficult to identify specific wavelengths via PLSR as it uses latent variables and components. SMLR highlights specific wavelengths that can be easily associated with chemical bonds and functional groups. R values (0.99) were higher in the PLSR model as compared to SMLR (R = 0.97) for both of the sample sets (calibration and validation). Descriptive statistics of TBC for the remaining 68 samples with predictive values are provided in Table 2. Furthermore, seven relevant wavelengths (Table 3) were ultimately identified using SMLR; they corresponded to the characteristic vibrations and stretching in protein, amide, and carbohydrate functional groups. Most of these wavelengths were in the second and third overtone regions. The wavelengths were 885.27 nm in the third overtone region, closer to the ArCH, CH_3_, and CH_2_ groups, highlighting the C–H third overtone; 1026.27 nm in the second and third overtone regions near RNH_2_, indicating the N–H stretching band of the second overtone; 1039.93 nm in the same region near the RNH_2_ and ArCH groups and indicating the N–H and C–H stretching bands of the second overtone region; 1068.38 nm, which is near 1100 nm and associated with ArCH groups, in the third and second overtone regions; and 1257.55 nm and 1267.75 nm in the second overtone region near the CH, CH_2_, and CH_3_ functional groups, indicating C–H stretching bands. Finally, 1453.59 nm is between 1300 nm and 1500 nm, corresponding to the first overtone region; it is closely associated with CONHR, CH_2_, CH, ROH, and CONH_2_. Bacterial metabolism involves the breakdown of organic compounds present in salmon fillets. As bacteria consume nutrients and produce metabolic byproducts, they can alter the chemical composition of the fillets. The various metabolic byproducts that are produced contain different bands, such as CH, OH, and NH; therefore, different wavelengths indicate changes in the chemical composition of salmon fillets as a result of bacterial spoilage. During spoilage, bacterial action or metabolites change fatty acid profiles and produce new compounds like exopolysaccharides (resulting in the stretching or shifting of C–H bands); spoilage by bacteria also involves protein degradation by microbial proteases (affecting N–H spectral signals), and aromatic rings (ArC–H) in different overtone regions also indicate bacterial enzymatic activity resulting in detectable changes in the ArC–H spectral bands. Moreover, changes in O–H bands represent changes in water content and the production of hydroxyl (OH)-rich metabolites, which increases during the spoilage process. Rodriguez-Saona et al. (2001) found out that N-acetylmuramic acid (a component of the peptidoglycan layer of bacterial cell walls) showed NIR bands (4432, 4355, 4152, and 4060 cm^−1^) quite similar to the bacterial suspensions used in their study [35], indicating the highly specific role of FT-NIR for the identification of bacterial signatures in order to enhance food safety standards. However, there are not sufficient literature studies that investigate the suitable wavelengths and optimal model via the application of FT-NIR for the determination of bacteria on raw salmon products in a nondestructive manner. Previous studies indeed highlighted the effectiveness of NIR with various other spectral imaging methods; for instance, a study on grass carp fillets has applied HSI with NIR to identify microbial spoilage and wavelength ranges [28]. Additionally, Cheng et al. (2015) investigated fish microbial spoilage in grass carp fillets using visible and near-infrared hyperspectral imaging (400–1000 nm), incorporating SPA-PLSR (successive projections algorithm partial least square regression) with an R^2^p of 0.90 and RMSEP of 0.5741 log_10_ CFU/g [28]. NIR-HSI with a wavelength range of 900 to 1700 nm was also applied to detect lactic acid bacteria (LAB) from salmon. They have incorporated least squares support vector machines to predict LAB values [36]. SWV-NIR (short-wavelength visible and NIR) indicated a wavelength range of 400 to 1000 nm can determine the freshness of chicken samples using a PLSR model with a prediction accuracy of a cross-validation coefficient (R^2^_cv_) of 0.82. They identified a few wavelengths as indicators (413, 426, 449, 460, 473, 480, 499, 638, 942, 946, 967, 970, and 982 nm) [37]. Furthermore, a tomato analysis was performed using NIR for the determination of soluble solid content [38]. NIR was also used to detect and quantify adulteration in honey [39]. Recently, emerging applications of NIR spectroscopy include the detection and differentiation of bacterial biofilms, highlighting its effectiveness and potential for future applications in food safety related to biofilm contamination [40]. With continuous advancements in NIR/FT-NIR technology, it presents a promising opportunity for exploring more convenient and invasive methods for bacterial analysis. In our study, we solely use FT-NIR with chemometric techniques to detect TBC, which highlights the cost-effective and less complex approach used in our methodology. Also, random cross-validation was conducted to test our models [18]. As per a review published in 2015, it was found that PLSR is the most widely used model for the analysis of spectra and microbial determination, and among the spectroscopic techniques, Vis/IR (visible/infrared) spectroscopy was applied more than 40 times [41]. However, we could not obtain suitable wavelengths by PLSR. We tried to investigate if models generated by SMLR are in consistent with PLSR, and proposed that results obtained with PLSR may be improved with a larger sample size to prevent the overfitting of data and to ensure the accuracy of model. A specific combination including FT-NIR with SNV and detrend preprocessing, followed by SMLR-dependent wavelength selection, in the determination of TBC on salmon fillets in a rapid, nondestructive manner highlights the novelty of our work and contribution to this emerging field.

### 3.3. Correlation Between Predicted and Laboratory Concentrations of TBC

**Figure 2:**
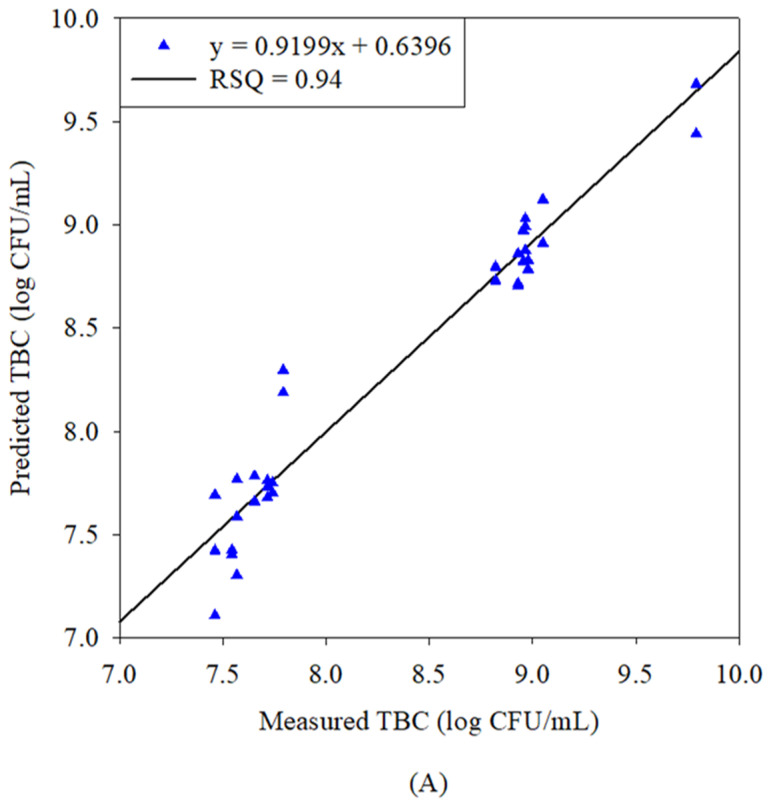

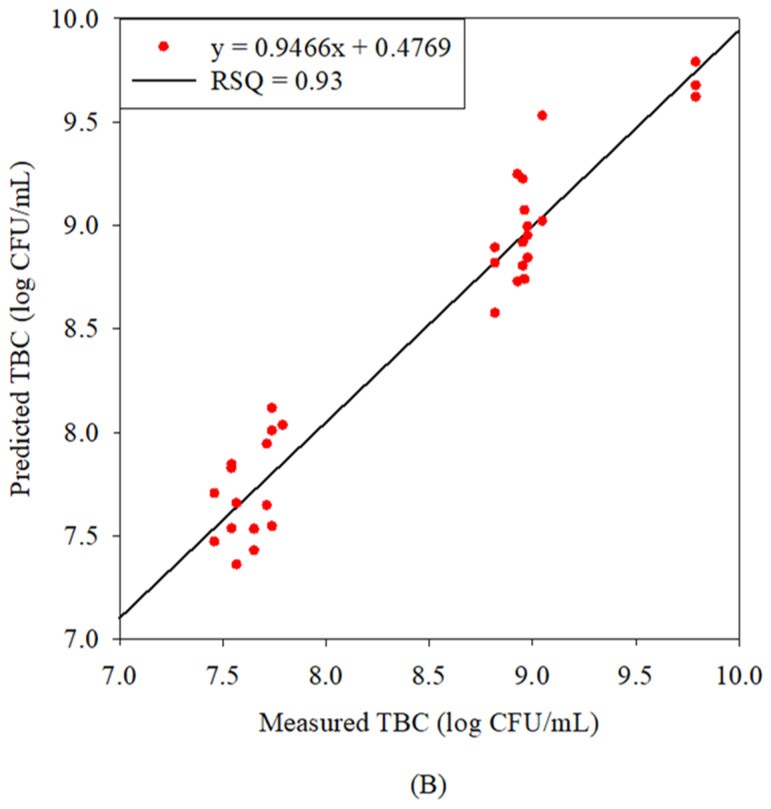
(**A**) Calibration (blue triangle points) and (**B**) validation (red circle points) graphs of measured versus predicted TBC obtained by SMLR model (in log CFU/mL).

Figure 2A,B present graphs of the measured (*x*-axis) and predicted (*y*-axis) TBCs for the calibration and validation sample sets, respectively. The measured TBCs were in the range of 7.69–9.68 and 7.70–9.78 log CFU/mL for the calibration and validation sample sets, respectively. For these sets, the predictive equation obtained by SMLR had coefficients of determination (R^2^) of 0.94 and 0.93, with an RMSE of 0.18 and 0.20 log CFU/mL, respectively. By PLSR, the predictive equations had an R^2^ of 0.98 and 0.99, with an RMSE of 0.10 and 0.06 log CFU/mL. However, in the previous literature, errors in the quantification models were in the range of 0.38 to 0.48 log CFU/g [42,43]. In our study, for the calibration set, the mean and STD were 8.342 and 0.754 log CFU/mL (measured TBC values), respectively; the corresponding values for the validation set were 8.429 and 0.762 log CFU/mL, respectively. Table 2 provides further information on descriptive statistics with regard to predictive TBC values obtained via the SMLR and PLSR models. The predicted and measured TBC values were thus found to be strongly correlated, and the resulting values had a lower RMSE, validating the efficacy of the proposed method. SMLR resulted in a lower RMSE in the calibration set as compared to the validation set, highlighting the better efficacy of the model for testing the data. The validation set built by the PLSR model resulted in a lower error, with an RMSE = 0.06 log CFU/mL, as compared to an RMSE = 0.10 log CFU/mL in the calibration set (Table 2). It indicates the insufficiency of the PLSR model to validate the results, because the higher-calibration RMSE value provides an idea that the model is overfitting the data. The RMSE value for the validation set should be higher or comparable with the calibration set. Previous studies highlighted the significance of NIR in the identification and quantification of bacteria from various food samples, including chicken [42], rainbow trout fillets [43], shredded cabbage [44], and on fish as well but with different approaches. They were able to demonstrate that NIR spectroscopy possesses the potential to discriminate between fresh and spoiled samples. In 2005, authors used FT-NIR technology for the differentiation among bacterial species and indicated that after applying soft independent modeling of class analogy (SIMCA) and PCA analysis in the region of 5100 to 4400 cm^−1^ (1960.78 nm to 2272.73 nm), the discrimination of bacterial species was possible [45]. The obtained spectral range was an indication of different bands like the C–H overtones of carbohydrates and lipids, as we also highlighted in our study. Moreover, FT-NIR has been used by many researchers for conducting studies on mycotoxin-contaminated agricultural products. The successful discrimination of contaminated moldy and uncontaminated figs (with a classification accuracy higher than 90%) was obtained using reflectance FT-NIRS [46]. Our methodology, including sample choice and chemometric approach, is different to the previous studies, hence it is not ideal to compare the wavelengths obtained in our study with those in the previous literature. This novel approach presents new insights that have not been previously investigated.

### 3.4. Analysis of Principal Components

**Figure 3:**
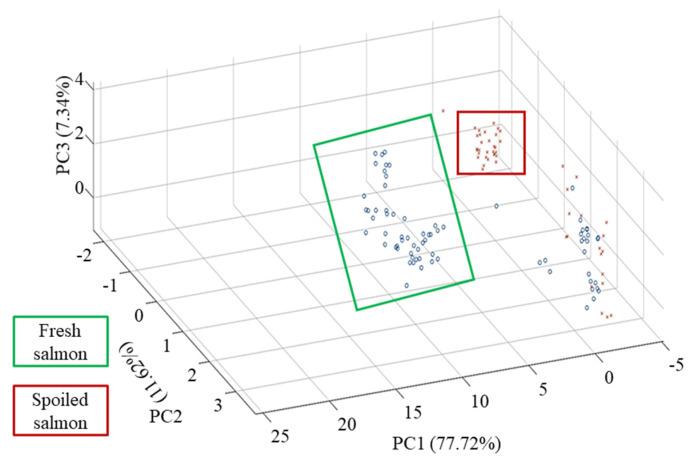
PCA representation of fresh versus spoiled salmon fillets.

A PCA analysis was conducted to compare the spectra obtained from fresh samples (day 0) and from those spoiled at room temperature for 4 days (day 4); it revealed various patterns in the dataset. The first three principal components (PC1, PC2, and PC3) collectively explained 96.68% of the total variance in the dataset, with PC1 contributing to the most variance (77.72%), followed by PC2 (11.62%) and PC3 (7.34%). A visual inspection of the PCA plot revealed the distinct clustering of the fresh and spoiled salmon samples along the PC1 and PC2 axes (Figure 3). The fresh samples were predominantly clustered in the positive region of PC1, indicating that there were similarities in their chemical compositions. The spoiled samples were distinctly clustered in the negative region of PC1. This clustering suggested significant differences in the chemical composition of the fresh and spoiled samples. The highlighted regions on the plot emphasize separation between the two groups; the spoiled and fresh samples are marked with red and green squares, respectively. However, some outliers were discovered. The salmon samples in both groups were from same store and same brand; however, the samples were not all procured contemporaneously. Moreover, raw salmon fillets cannot be completely free from bacteria. Lipids (fats) and carbohydrates are common constituents in salmon samples and bacteria, due to which FT-NIR spectra may overlap, resulting in unclear segregation in the FT-NIR results. To mitigate this limitation in a future study, bacterial growth and enzymatic changes could be inhibited in reference samples to enable a better discrimination between groups. The model generated in this study resulted in good correlation values with low errors, highlighting the significance of FT-NIR in providing the necessary information, which could be obtained after applying preprocessing and chemometric techniques. This study provides a benchmark for novel methodologies in future studies attempting to identify seafood spoilage in a non-invasive manner, especially for fish. With an advanced chemometric approach, FT-NIR spectroscopy could be a reliable tool for quality and safety assessments in the seafood industry.

## 4. Conclusions

The integration of FT-NIR spectroscopy with SMLR effectively identified bacterial spoilage (TBC) in salmon. This method had a high predictive accuracy, with R values of 0.970 in both the calibration and validation sets and with an acceptable RMSE of 0.18 and 0.20 log CFU/mL, respectively. This indicates a robust correlation between predicted and referenced values, particularly for the TBC. The wavelengths crucial to the identification of spoilage were also identified and can be used as valuable inputs for the development of reference models. Moreover, our study indicates that increasing sample sizes to further strengthen the reliability and applicability of the models is critical, especially with the PLSR model. Additionally, PCA revealed that fresh and spoiled salmon samples could be distinguished by their chemical composition. The first three PCs explained 96.68% of the variance, highlighting the robustness of the proposed analytical approach. Hence, the acquisition of key wavelengths is a promising method for establishing nondestructive reference models. The calibration and validation models developed in this study were effective for predicting the TBC in raw salmon samples. Moreover, this is an innovative approach showing promise for potential applications in the food industry due to FTNIR’s rapid and nondestructive characteristics. For food industries, it offers real-time, on-site quality assessment and helps minimize spoilage-related economic losses. However, the methodology must be validated for diverse samples and bacterial strains. A comprehensive approach for evaluating various sea food products with reference to a database of many bacterial groups is required. The current model is based on naturally spoiled samples in a controlled temperature range, which may affect its generalizability. The spoilage process varies and depends on various factors including fish species, storage conditions, and type of microorganisms. To address these limitations, future research should focus on varied storage environments, temperature conditions, and types of microorganisms. Additionally, to further validate the model’s performance and to determine its sensitivity in identifying the lowest detectable levels of spoilage, studies with controlled inoculation are needed. This study contributes to the body of knowledge and encourages researchers to explore improved methodologies for the advancement of safe fisheries and aquaculture systems.

## Figures and Tables

**Table 1:** Optimal parameters and results of SMLR and PLSR models to detect TBC in raw salmon fillets.

Constituent	Model	Data Processing	Spectral Range (nm)	Segment	Derivative Gap	RC	RV
TBC	SMLR	SNV and Detrend-d^1^	867–2505	2	2	0.97	0.97
TBC	PLSR	SNV and Detrend-d^1^	867–2505	2	2	0.99	0.99

RC, correlation in calibration set; RV, calibration in validation set; PLSR, partial least square regression; SMLR, stepwise multiple linear regression SNV, standard normal variate; d^1^, first derivative.

**Table 2:** Descriptive statistics of the TBC (predicted values as log CFU/mL) in the calibration and validation sample sets.

Model	Sample Set	n	Range (TBC log CFU/mL) Predicted	Mean (log CFU/mL)	STD (log CFU/mL)	RMSE (log CFU/mL)
**SMLR**	Calibration	34	7.69–9.68	8.31	0.72	0.18
**SMLR**	Validation	34	7.70–9.79	8.43	0.76	0.20
**PLSR**	Calibration	34	7.44–9.82	8.34	0.75	0.10
**PLSR**	Validation	34	7.48–9.81	8.40	0.77	0.06

n, number of samples; TBC, total bacterial count; Predicted, predicted values via model; CFU, colony forming unit; STD, standard deviation; RMSE, root mean square error; PLSR, partial least square regression; SMLR, stepwise multiple linear regression.

**Table 3:** Wavelengths indicative of spoilage by TBC (obtained via SMLR) in raw salmon fillets.

Wavelength	885.27 nm (11,294.2 cm^−1^)	1026.27 nm (9740.3 cm^−1^)	1039.93 nm (9616.8 cm^−1^)	1068.38 nm (9360.9 cm^−1^)	1257.55 nm (7953.0 cm^−1^)	1267.75 nm (7886.2 cm^−1^)	1453.49 nm (6878.6 cm^−1^)
FT-NIR absorption band region	Third overtone	Second overtone	Second overtone	Second overtone	Second overtone	Second overtone	First overtone
Indication	ArCH, CH3 and CH2	NH	RNH2, ArCH	ArCH	CH, CH2, CH3	CH	CONHR, CH2, CH, ROH, H2
Significance	C–H	N–H	N–H, C–H	C–H	C–H	C–H	O–H, N–H, C–H

TBC, total bacterial count; cm, centimeter; nm, nanometer.

## Data Availability

The original contributions presented in this study are included in the article. Further inquiries can be directed to the corresponding author.
